# The SNARE Protein Syp71 Is Essential for Turnip Mosaic Virus Infection by Mediating Fusion of Virus-Induced Vesicles with Chloroplasts

**DOI:** 10.1371/journal.ppat.1003378

**Published:** 2013-05-16

**Authors:** Taiyun Wei, Changwei Zhang, Xilin Hou, Hélène Sanfaçon, Aiming Wang

**Affiliations:** 1 Southern Crop Protection and Food Research Centre, Agriculture and Agri-Food Canada, London, Ontario, Canada; 2 Department of Biology, The University of Western Ontario, London, Ontario, Canada; 3 State Key Laboratory of Crop Genetics and Germplasm Enhancement, Nanjing Agricultural University, Nanjing , People's Republic of China; 4 Pacific Agri-Food Research Centre, Agriculture and Agri-Food Canada, Summerland, British Columbia, Canada; University of Kentucky, United States of America

## Abstract

All positive-strand RNA viruses induce the biogenesis of cytoplasmic membrane-bound virus factories for viral genome multiplication. We have previously demonstrated that upon plant potyvirus infection, the potyviral 6K2 integral membrane protein induces the formation of ER-derived replication vesicles that subsequently target chloroplasts for robust genome replication. Here, we report that following the trafficking of the *Turnip mosaic potyvirus* (TuMV) 6K2 vesicles to chloroplasts, 6K2 vesicles accumulate at the chloroplasts to form chloroplast-bound elongated tubular structures followed by chloroplast aggregation. A functional actomyosin motility system is required for this process. As vesicle trafficking and fusion *in planta* are facilitated by a superfamily of proteins known as SNAREs (soluble *N*-ethylmaleimide-sensitive-factor attachment protein receptors), we screened ER-localized SNARES or SNARE-like proteins for their possible involvement in TuMV infection. We identified Syp71 and Vap27-1 that colocalize with the chloroplast-bound 6K2 complex. Knockdown of their expression using a *Tobacco rattle virus* (TRV)-based virus-induced gene silencing vector showed that Syp71 but not Vap27-1 is essential for TuMV infection. In *Syp71*-downregulated plant cells, the formation of 6K2-induced chloroplast-bound elongated tubular structures and chloroplast aggregates is inhibited and virus accumulation is significantly reduced, but the trafficking of the 6K2 vesicles from the ER to chloroplast is not affected. Taken together, these data suggest that Syp71 is a host factor essential for successful virus infection by mediating the fusion of the virus-induced vesicles with chloroplasts during TuMV infection.

## Author Summary

Potyviruses constitute the largest group of known plant viruses which includes many agriculturally important viruses. Like all other positive-strand RNA viruses, potyviruses induce the cytoplasmic membranous-bound virus factory for viral genome multiplication. But the mechanism by which such a factory is formed and associated with cellular membranes remains elusive. Recently we have demonstrated that upon potyvirus infection, the potyviral 6K2 integral membrane protein is responsible for inducing the formation of the ER-derived replication vesicles that subsequently target chloroplasts for robust genome replication. Here, we report that following the trafficking of the TuMV 6K2 vesicles to chloroplasts, 6K2 vesicles accumulate at the chloroplasts to form chloroplast-bound elongated tubular structures followed by chloroplast aggregation. We show that a host protein, Syp71, is associated with the 6K2 vesicles. When the *Syp71* gene is silenced, TuMV infection is inhibited and the formation of the chloroplast-bound 6K2 complex is arrested but the trafficking of the 6K2 vesicles from the ER to chloroplast is not affected. Therefore, Syp71 is a host factor that mediates the fusion of 6K2 vesicles with chloroplasts during TuMV infection. Our study sheds new light onto the mechanism underlying the association of potyviral 6K2 vesicles with chloroplasts.

## Introduction

The host endomembrane system directly contributes to the formation of virus-induced membrane-bound virus factory for positive-strand RNA virus replication [1], [2]. Depending on the virus, membranes from distinct cellular organelles such as the endoplasmic reticulum (ER), chloroplast, mitochondrion, endosome, and peroxisome are recruited to house the virus factory [3]. In the past decade, efforts have been made to elucidate the molecular mechanisms underlying the assembly of the virus factory with cellular membranes. For instance, genome-wide screens for host factors affecting *Tomato bushy stunt virus* (TBSV) replication in yeast, has led to the identification of seven ESCRT (endosomal sorting complex required for transport) proteins involved in the assembly of membrane-bound replicase complexes through interaction with the viral integral membrane protein p33 [4]–[6]. The characterization of *Arabidopsis* mutants defective in *Tobacco mosaic virus* (TMV) infection revealed several host integral membrane proteins, including the Tom1/3 proteins, the putative membrane anchor for the virus factory, and the Tom2 protein, which most likely plays an accessory role in replication [7], [8]. The host vesicle associated protein (VAP) was suggested to be involved in the biogenesis of membrane vesicles induced by the 60K protein of *Cowpea mosaic virus* (CPMV) [9]. The VAP protein interacts with the CPMV 60K protein and colocalizes with the ER-derived vesicles that contain the 60K protein in CPMV-infected plant cells [9]. The second 6,000-molecular-weight protein (designated 6K2 or 6K) of the family *Potyviridae*, the largest and most agriculturally important family of plant positive-strand RNA viruses, is an integral membrane protein that induces the formation of 6K2-containing membranous vesicles at ER exit sites [10], [11]. The 6K2-induced vesicles subsequently target chloroplasts to form chloroplast-6K2 complexes that harbour the virus factory for potyvirus replication [12].

In eukaryotic cells, specific membrane fusion between transport vesicles and target membranes is mediated by the SNARE (soluble *N*-ethyl-maleimide-sensitive-factor attachment protein receptors) complex that assembles into a tight cluster of four coiled-coil helices [13]–[16]. In *Arabidopsis thaliana*, at least 64 SNARE proteins have been identified and classified into the Qa-, Qb-, Qc-, and R-groups [13], [17]. Of the ternary SNARE complexes identified in plant cells thus far, the Qa-PEN1/Qb+Qc-SNAP33/R-VAMP721(722) complex functions in disease resistance and the Qa-SYP22/Qb-SYP51/Qc-VTI11/R-VAMP727 complex operates in seed development [18], [19]. Previous reports showed that the ER-localized SNARE molecules contain SYP81/AtUfe1 (Qa), SYP71, SYP72, and SYP73 (Qc), AtSec22 and AtVAMP723 (R) in *Arabidopsis* [13]. Furthermore, *Arabidopsis* has 10 VAPs grouped in the VAP33 subfamily of SNAREs which also localize on the ER membrane [20]. These ER-localized SNARE or SNARE-like molecules might selectively function in mediating ER-associated vesicular trafficking, docking and fusion.

Due to the fact that the chloroplast-bound 6K2 vesicles housing the virus factory of potyviruses are of ER origin, it is possible that ER-localized SNARE or SNARE-like proteins play a role in the formation of the 6K2–chloroplast complex. In the present study, we show that following the trafficking of 6K2 to chloroplasts, 6K2 induces the formation of chloroplast aggregates and elongated tubular structures at the junction between adjacent chloroplasts. We demonstrate that two ER-localized SNAREs, i.e., Qc-Syp71 and Vap27-1, colocalize with the chloroplast-bound 6K2 structure. We further identify Syp71 as a SNARE essential for virus infection via mediating the formation of 6K2-induced elongated tubular structures and chloroplast clumps in infected plants.

## Results

### The ER-derived 6K2 vesicles translocate to chloroplasts and form elongated tubular structures between adjacent chloroplasts

**Figure 1:**
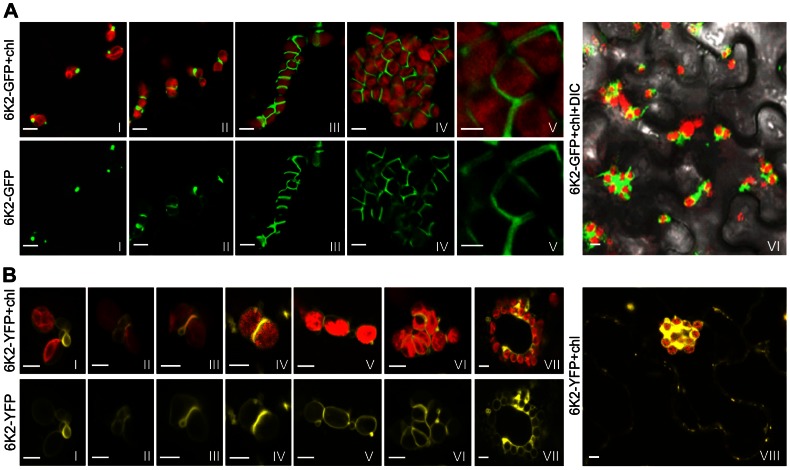
Formation of chloroplast-bound elongated tubular structures and chloroplast aggregates in ***N.*** *benthamiana* during TuMV infection. (A) 6K2-GFP targeted chloroplasts, formed elongated tubular structures at the junctions between adjacent chloroplasts and induced the formation of chloroplast clumps in *N. benthamiana* leaves infected by a recombinant TuMV clone (TuMV::6K2-GFP) containing a GFP-tagged 6K2. Frame I, 48 hr post infiltration (hpi); frame II, 72 hpi; frames III and IV, 96 hpi; frame V, an enlarged view of part of frame IV; frame VI, a lower magnification image, 96 hpi Upper panel shows both the GFP fluorescence (in green) and the chloroplast autofluorescence (Chl, in red). Lower panels only show the GFP signal. (B) 6K2-YFP induced the formation of chloroplast-bound elongated tubular structures and chloroplast aggregates in *N. benthamiana* leaves expressing 6K2-YFP alone. Frame I, 48 hpi; frames II–IV, 60 hpi; frames V–VII, 72 hpi; frame VIII, a lower magnification image. The YFP fluorescence (in yellow) and the chloroplast autofluorescence (in red) are shown in the upper panel, while only the YFP signal is shown in the lower panel. Bars, 8 µm.

Previous studies showed that the potyviral 6K2 membrane protein induces the formation of ER-derived vesicles [10] and potyviral infection can induce the formation of chloroplast aggregates [21]. Recently, we reported that the ER-derived 6K2-containing vesicles in TuMV-infected leaves translocate from the ER to chloroplasts for potyviral genome replication [11], [12]. To investigate if 6K2 vesicles are involved in chloroplast aggregations, a recombinant TuMV (TuMV::6K2-GFP) that contains a green fluorescent protein (GFP)-tagged 6K2 was introduced into *Nicotiana benthamiana* leaf cells via agroinfiltration. At 48 hrs post infiltration (hpi), 6K2-GFP vesicles were found at the periphery of chloroplasts (Fig. 1A, frame I), consistent with our previous observation [12]. At 72 hpi, chloroplast aggregation occurred in about 50% infected cells and 6K2-GFP formed an elongated tubular structure at the junctions between two adjacent chloroplasts (Fig. 1A, frame II). At 96 hpi, more chloroplast aggregates (>85% of infected cells) were observed (Fig. 1A, frames III–VI). Typically, about 20 chloroplasts were tightly apposed to each other in a chain or irregularly grouped, and elongated tubular structures highlighted by 6K2-GFP were found between two adjacent chloroplasts (Fig. 1A, frame III). Occasionally, large chloroplast clumps, consisting of up to 50 chloroplasts, were found (Fig. 1A, frame IV). In such clumps, chloroplasts were irregularly grouped but closely apposed to each other to form a spherical amorphous mass. At higher magnifications, an elongated tubular structure enveloped by 6K2-GFP was evident between adjacent chloroplasts in the aggregates (Fig. 1A, frame V).

To determine the distribution of 6K2 vesicles in the absence of viral infection, the yellow fluorescent protein tagged 6K2 (6K2-YFP) was transiently expressed in *N. benthamiana* leaf cells via agroinfiltration. When expressed alone, at 48 hpi, small ring-like vesicles of 6K2-YFP initially accumulated between two closely located, but physically separate chloroplasts (Fig. 1B, frame I). Intriguingly, these vesicles subsequently coalesced, leading to the formation of a bridge across the outer envelopes of the two adjacent chloroplasts (Fig. 1B, frame II). The vesicle bridge apparently promoted the adhesion of the adjacent chloroplasts, with a strong labelling of 6K2-YFP at the junctions (Fig. 1B, frames III and IV), similar to elongated tubular structures induced by 6K2-GFP during viral infection (Fig. 1A, frames II–IV). After 72 hpi, 6K2-YFP induced chloroplast aggregates (Fig. 1B, frames V–VIII). These data therefore demonstrate that the 6K2 protein is able to induce the formation of elongated tubular structures between adjacent chloroplasts independently of any other TuMV-encoded proteins or viral RNA replication.

### The actomyosin motility system mediates the formation of chloroplast-bound 6K2 elongated tubular structures and chloroplast aggregates

**Figure 2:**
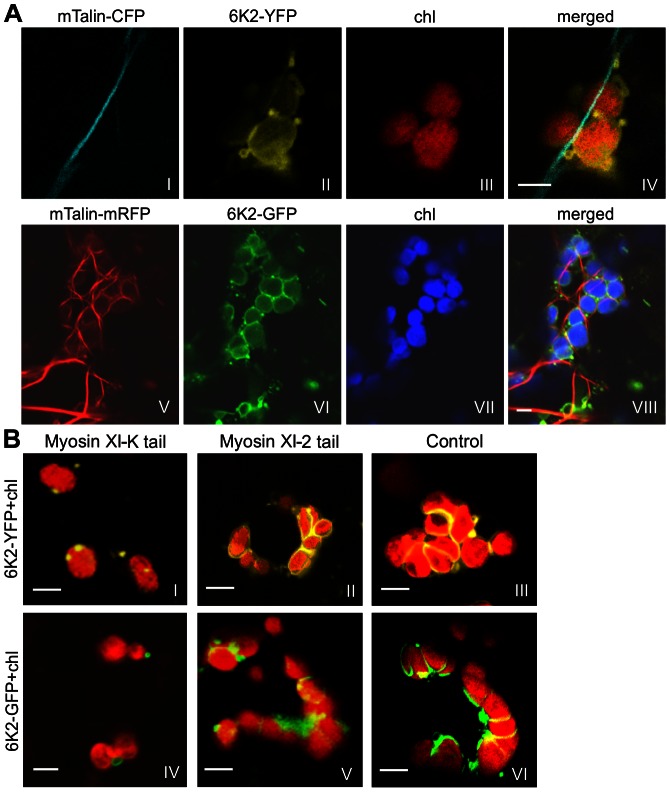
The actomyosin motility system mediates the formation of 6K2-induced elongated tubular structures connecting adjacent chloroplasts. (A) Coalignment of 6K2 vesicles on the actin microfilaments labelled by mTalin-CFP or mTalin-mRFP. Frames I through IV, 6K2-YFP was transiently expressed with mTalin-CFP in *N. benthamiana* leaves. Chl, chloroplast. Frames V through VIII, mTalin-mRFP was transiently expressed in *N. benthamiana* leaves infected by TuMV::6K2-GFP. (B) The effects of expression of the myosin XI-K tails, a dominant-negative mutant of XI-K or XI-2, on the formation of chloroplast aggregates induced by the 6K2 protein. Frames I–III, 6K2-YFP was transiently expressed alone. Frames IV–VI, 6K2-GFP was expressed in the presence of TuMV infection (using the TuMV::6K2-GFP infectious clone). Bars, 8 µm.

Actin filaments and myosin XI may play a role in the mobility of chloroplasts through direct interactions with the chloroplast outer envelope membrane [22], [23]. We observed colocalization between actin filaments stained by mTalin-CFP and the elongated tubular structure induced by 6K2-YFP (in the absence of viral infection; Fig. 2A, frames I–IV) or 6K2-GFP (in the presence of viral infection; Fig. 2A, frames V–VIII) at the junctions between adjacent chloroplasts in *N. benthamiana* leaf cells (Fig. 2A). The potential role of myosin XI-K in the formation of elongated tubular structures induced by the 6K2 protein was assayed using the myosin XI-K tail, a dominant negative mutant of myosin XI-K [24]. Overexpression of the myosin XI-K tail dramatically inhibited the formation of the 6K2-induced elongated tubular structures at the junction of adjacent chloroplasts as well as chloroplast aggregates (Fig. 2B, frames I and IV), either in the absence of viral infection (indicated as 6K2-YFP) or in the presence of viral infection (indicated as 6K2-GFP). Such inhibition was not seen in the *N. benthamiana* leaf cells overexpressing the myosin XI-2 tail (Fig. 2B, frames II and V) or in the negative control leaf cells (Fig. 2B, frames III and VI). Taken together, these data indicate that the formation of chloroplast-bound 6K2 elongated tubular structures and chloroplast aggregates requires the functional actomyosin motility system.

### Syp71 and Vap27-1 are recruited to the 6K2-induced elongated tubular structure

**Figure 3:**
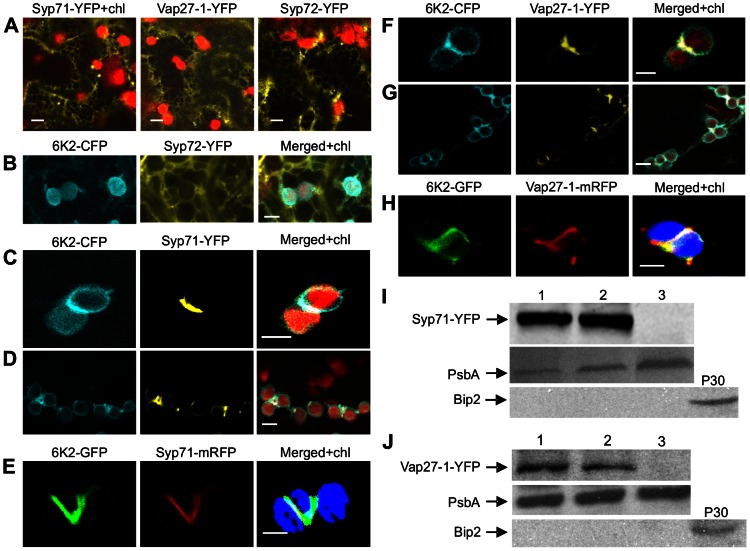
Syp71 and Vap27-1 are recruited to the 6K2-induced elongated tubular structure. (A) Localization of Syp71-YFP, Vap27-1-YFP and Syp72-YFP. Syp71-YFP (frame I), Vap27-1-YFP (frame II) and Syp72-YFP (frame III) were transiently expressed alone in *N. benthamiana* leaves. (B) 6K2-CFP and Syp72-YFP were transiently coexpressed in *N. benthamiana* leaves. (C and D) Syp71-YFP (yellow signal) was recruited to the elongated tubular structure containing 6K2-CFP (blue signal) at the junction between clumped chloroplasts (red autofluorescence) when they were coexpressed in *N. benthamiana*. (E) Syp71-mRFP (red signal) was recruited to the elongated tubular structure containing 6K2-GFP (green signal) at the junction between adjacent chloroplasts (autofluorescence signal was color-coded in blue) in *N. benthamiana* leaves infected by TuMV 6K2::GFP. (F and G) Vap27-1-YFP (yellow signal) was recruited to the elongated tubular structure containing 6K2-CFP (blue signal) at the junction between clumped chloroplasts (red autofluorescence) when 6K2-CFP and YFP-Syp71 were coexpressed in *N. benthamiana*. (H) Vap27-1-mRFP (red signal) was recruited to the elongated tubular structure containing 6K2-GFP (green signal) at the junction between adjacent chloroplasts (color-coded in blue) in *N. benthamiana* leaves infected by TuMV 6K2::GFP. Bars in (A through H), 8 µm. (I) Western blot analysis confirmed that Syp71-YFP was associated with chloroplasts when coexpressed with 6K2-CFP or during TuMV infection. The filters were detected by Syp71 antibodies (top panel), PsbA antibodies (middle panel) and Bip2 antibodies (bottom panel). 1, chloroplasts were purified from *N. benthamiana* leaves coexpressing Syp71-YFP and 6K2-CFP; 2, from leaves expressing Syp71-YFP and infected with TuMV::6K2-GFP; 3, from leaves expressing Syp71-YFP; P30, crude membrane fraction of leaves from mock-treated plants. PsbA and Bip2 serve as chloroplast and ER markers, respectively. (J) Western blot analysis confirmed that Vap27-1-YFP was associated with chloroplasts when coexpressed with 6K2-CFP or during TuMV infection. The filters were detected by Vap27-1 antibodies (top panel), PsbA antibodies (middle panel) and Bip2 antibodies (bottom panel). 1, chloroplasts were purified from *N. benthamiana* leaves coexpressing Vap27-1-YFP and 6K2-CFP; 2, from leaves expressing Vap27-1-YFP and infected with TuMV::6K2-GFP; 3, from leaves expressing Vap27-1-YFP; P30, crude membrane fraction.

As the initial biogenesis of membranous vesicles of 6K2 occurs at ER exit sites (ERES) on the ER membrane [11], [12], their translocation may require proteins from the SNARE family that mediates specific membrane fusion between transport vesicles and target membranes. To determine if SNARE proteins are involved in the translocation of the 6K2 vesicles, we first cloned seven putative ER-localized SNAREs or SNARE-like proteins from *Arabidopsis* including Syp71, Syp72, Syp73, Syp81, VAM723, Vap27-1 and Vap27-2 and constructed plant expression vectors for the transient expression of these SNARE proteins tagged with YFP. As expected, expression of each of these proteins alone revealed a typical ER reticulated pattern (Fig. 3A and data not shown). When each of these SNARE-YFP fusion proteins was transiently coexpressed with 6K2-CFP (cyan fluorescent protein), only Syp71-YFP and Vap27-1-YFP preferentially colocalized to 6K2-induced elongated tubular structures at the junctions of adjacent chloroplasts (Fig. 3C, D, F, G). Other SNARES tested such as Syp72 did not colocalize with the 6K2-induced structure (Fig. 3B). To investigate if Syp71 and Vap27-1 are recruited to the 6K2-induced elongated tubular structures during viral infection, Syp71-mRFP (monomeric red fluorescent protein) or Vap27-1-mRFP was coinfiltrated into *N. benthamiana* with the recombinant TuMV (TuMV::6K2-GFP). At 72 hpi, the fluorescence of Syp71-mRFP or Vap27-1-mRFP colocalized with the chloroplast-bound 6K2-GFP elongated tubular structures (Fig. 3E, H). To confirm the association of Syp71 or Vap27-1 with the chloroplast, chloroplasts were purified from *N. benthamiana* leaves coinfiltrated with the following different combinations: (1) 6K2-CFP and Syp71-YFP (or Vap27-1-YFP); (2) the TuMV::6K2-GFP infectious clone and Syp71-YFP (or Vap27-1-YFP); and (3) Syp71-YFP or Vap27-1-YFP alone. Western blotting experiments were carried out to detect Syp71 and Vap27-1 in the purified chloroplasts with antisera against Syp71 or Vap27-1. A protein corresponding to the predicted size for Syp71-YFP was detected by Syp71 antibodies in the chloroplasts purified from the leaves coexpressing Syp71-YFP and 6K2-CFP or expressing Syp71-YFP in the presence of TuMV infection (TuMV::6K2-GFP) (Fig. 3I, lanes 1 and 2). Similarly, a protein with the predicted size for Vap27-1-YFP was also found in the chloroplasts purified from the leaves coexpressing Vap27-1-YFP and 6K2-CFP or expressing Vap27-1-YFP in the presence of TuMV infection (TuMV::6K2-GFP) (Fig. 3J, lanes 1 and 2). In contrast, no detectable Syp71-YFP or Vap27-1-YFP was evident in chloroplasts purified from the leaves expressing Syp71-YFP or Vap27-1-YFP alone (Fig. 3I and J, lane 3). The purity of chloroplasts was confirmed by immunoblotting to detect chloroplast protein PsbA and ER marker Bip2 (Fig. 3I and J). These data suggest that Syp71 or Vap27-1 are tightly associated with chloroplasts when coexpressed with 6K2-CFP or in the presence of viral infection with TuMV::6K2-GFP and that the association of Syp71 or Vap27-1 with chloroplasts is mediated by the 6K2 protein.

### Syp71 does not directly interact with the 6K2 protein

**Figure 4:**
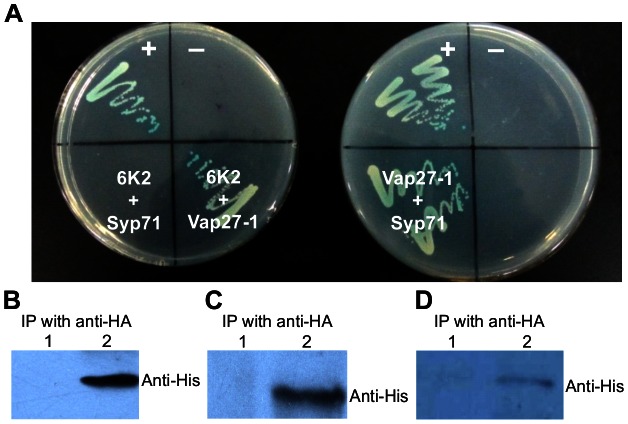
Syp71 does not interact directly with the TuMV 6K2. (A) Yeast two-hybrid assay of protein-protein interactions among the TuMV 6K2, Syp71 and Vap27-1 proteins. The transformants were plated on an SD/-Leu/-Trp/-His/-Ade medium. Left plate: +, positive control (pBT3-STE+pOst1-NubI); − , negative control (pBT3-STE-6K2+pPR3-N); 6K2+Syp71, pBT3-STE-6K2+pPR3-N-syp71; 6K2+Vap27-1, pBT3-STE-6K22+pPR3-N-vap27-1. Right plate: +, positive control (as above); −, negative control (pBT3-STE-vap27-1+pPR3-N); Vap27-1+Syp71, pBT3-STE-vap27-1+pPR3-N-syp71. (B) 6K2 interacted with Vap27-1 in *N. benthamiana* leaves. After removal of tissues and cell debris, the cleared lysate was immunoprecipitated with HA antibodies, and the immunoprecipitated proteins were detected by His antibodies. 1, immunoprecipitation protein samples from leaves expressing Vap27-1-His alone; 2, from leaves coexpressing 6K2-HA and Vap27-1-His. (C) Syp71 interacted with Vap27-1 in *N. benthamiana* leaves. The cleared lysate was immunoprecipitated with HA antibodies, and the immunoprecipitated proteins were detected by His antibodies. 1, immunoprecipitation protein samples from leaves expressing Vap27-1-His; 2, from leaves coexpressing Syp71-HA and Vap27-1-His. (D) Syp71, 6K2 and Vap27-1 were coimmunoprecipitated when they were transiently coexpressed in *N. benthamiana* leaves. The cleared lysate was immunoprecipitated with HA antibodies, and the immunoprecipitated proteins were detected by His antibodies. 1, immunoprecipitation protein samples from leaves coexpressing Vap27-1, 6K2 and Syp71-His; 2, from leaves coexpressing Syp71-His, 6K2-HA and Vap27-1.

To explore how Syp71 and Vap27-1 are recruited by the 6K2 protein, the DUAL membrane system, a variant of the yeast two-hybrid assay, was used to determine if there are any protein-protein interactions among the three membrane proteins 6K2, Syp71 and Vap27-1. Our results showed that 6K2 interacted with Vap27-1, but not with Syp71 (Fig. 4A). Syp71 was also found to bind to Vap27-1 (Fig. 4A). To further confirm these interactions, 6K2, Syp71 and Vap27-1 were fused with a His or HA tag and the resulting fusion proteins were transiently expressed in *N. benthamiana* leaves in different combinations, followed by immunoprecipitation. As shown in Fig. 4B and C, positive interactions were confirmed between 6K2-HA and Vap27-1-His and between Syp71-HA and Vap27-1-His. Only a weak interaction signal was detected between 6K2-HA and Syp71-His, when these proteins were co-expressed with the untagged Vap27-1 (Fig. 4D). These data suggest that Syp71 is not likely directly recruited by 6K2, and the association of Syp71 with the chloroplast-bound 6K2-induced tubular structures may be via a third protein.

### Downregulation of the expression of Syp71 in *Arabidopsis* inhibits TuMV infection

**Figure 5:**
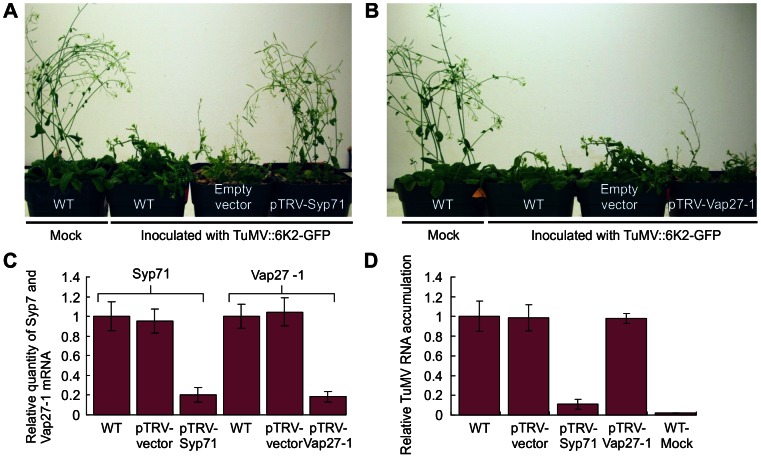
The requirement of Syp71 for TuMV infection. (A) Knockdown of *Syp71* expression inhibited TuMV infection in *Arabidopsis*. pTRV1 and pTRV2-Syp71 or vector controls were agroinfiltrated into *Arabidopsis*. After 10 days post infiltration, seedlings were inoculated with TuMV::6K2-GFP. Photographs were taken 12 days post TuMV inoculation. (B) Knockdown of Vap27-1 expression did not significantly affect TuMV infection in *Arabidopsis*. pTRV1 and pTRV2-Vap27-1 or vector controls were agroinfiltrated into *Arabidopsis*. After 10 days post infiltration, seedlings were inoculated with TuMV::6K2-GFP. Photographs were taken 12 days post TuMV inoculation. (C) Relative expression levels of Syp71 and Vap27-1 in *Arabidopsis* plants inoculated with TRV-based VIGS vectors targeting *Syp71* or *Vap27-1*. qPCR assays were conducted to quantify Syp71 and Vap27-1 mRNAs 12 days post agroinfiltration. Wild type (mock-inoculated) and plant infected by empty pTRV vectors were used as controls. Three independent experiments, each consisting of three replicates, were carried out for quantification analyses. The values were normalized against *ActinII* transcripts in the same sample. The values represent means of fold change relative to the wild type. Error bars represent standard deviations. (D) Relative TuMV RNA accumulation in *Syp1*- and *Vap27-1*-downregulated or control *Arabidopsis* plants. qPCR assays were conducted to quantify the TuMV viral RNA 10 days post TuMV inoculation. Wild type and plants infected by empty pTRV vectors were used as controls. Three independent experiments, each consisting of three replicates, were carried out for quantification analyses. The values were normalized against *ActinII* transcripts in the same sample. The values represent means of fold change relative to the wild type. Error bars represent standard deviations.

To investigate whether 6K2-associated proteins Syp71 and Vap27-1 are required for TuMV infection, virus induced gene silencing (VIGS) was used to knock down the expression of *Syp71* and *Vap27-1*. A cDNA fragment of *Syp71* or *Vap27-1* was cloned into the RNA2-derived vector of *Tobacco rattle virus* (TRV). The obtained TRV-Syp71 or TRV-Vap27-1 vectors were co-introduced into *Arabidopsis* with the TRV1 vector via agro-infiltration [25]. Real-time PCR analysis confirmed that *Syp71* or *Vap27-1* mRNA levels in the treated plants decreased by 85% compared to the negative control (plants infected with the TRV1 and TRV2 empty vectors) at 12 days post agro-infiltration (Fig. 5C). *Vap27-1*-downregulated *Arabidopsis* plants did not show detectable developmental defects or other phenotypes in comparison with the control plants. *Syp71*-downregulated *Arabidopsis* plants were slightly smaller in stature and the upper new rosette leaves were also slightly smaller. The most drastic effect was that the *Syp71*-silenced plants produced only a few seeds and these seeds were not viable, suggesting that Syp71 may be important for embryogenesis. New leaves from the non-silenced wild type *Arabidopsis* plants (either treated with buffer or pTRV2 empty vector), and *Syp71*- and *Vap27-1*-downregulated plants were inoculated with TuMV::6K2-GFP. After 10 days post inoculation, typical TuMV-induced symptoms such as mosaic and dwarfism were observed in wild type or pTRV2 empty vector-treated plants (Fig. 5A). *Vap27-1*-downregulated plants also showed similar symptoms (Fig. 5B). In contrast, *Syp71*-downregulated plants inoculated with TuMV were slightly shorter in stature than the control plants but did not show other noticeable typical TuMV symptoms (Fig. 5A). Real-time RT-PCR was carried out to quantify TuMV viral RNA. The TuMV viral RNA decreased about 10 times in *Syp71-*silenced plants when compared to the non-silenced control plants treated with the empty VIGS vector or with the inoculation buffer (mock) (Fig. 5D). No significant difference in the concentration of TuMV viral RNA was found between the *Vap27-1*-downregulated plants and the control plants (Fig. 5D). These data suggest that suppression of *Syp71* expression effectively inhibits TuMV infection in *Arabidopsis*, while down-regulation of *Vap27-1* does not.

### Syp71 is essential for TuMV replication and the formation of the chloroplast-bound 6K2 elongated tubular structures and chloroplast clumps

**Figure 6:**
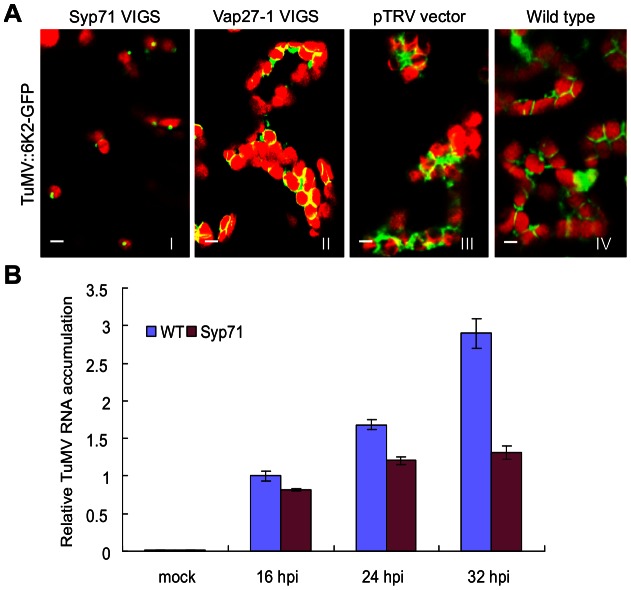
Downregulation of *Syp71* expression prevents the formation of chloroplast-bound elongated tubular structures and chloroplast aggregation and inhibits virus accumulation *in planta*. (A) Downregulation of *Syp71* expression rather than *Vap27-1* expression compromised the formation of chloroplast-bound elongated tubular structures and chloroplast aggregation in *N. benthamiana*. Frame I, *Syp71*-downregulated plants; frame II, *Vap27-1*-downregulated plants; frame III, control plants treated with pTRV empty vectors; frame IV, wild type plants (mock treated). Images were taken in the inoculated leaves 72 hrs post agroinfiltration with TuMV::6K2-GFP. Bars, 8 µm. (B) TuMV RNA accumulation in wild type or *Syp71*-downregulated *Arabidopsis* protoplasts. qRT-PCR was used to quantify TuMV RNA accumulation in protoplasts at indicated time points. Three independent experiments, each consisting of three replicates, were carried out for quantification analyses. The values were normalized against *ActinII* transcripts in the same sample. The values represent means of fold change relative to the wild type at 16 hours post inoculation (hpi) with TuMV. Error bars represent standard deviations.

To determine if downregulation of *Syp71* or *Vap27-1* expression affects the formation of chloroplast-bound 6K2 tubular structures, *N. benthamiana* plants were treated with the TRV-based VIGS vectors to knock down *Syp71* and *Vap27-1* expression. The *N. benthamiana* plants were then inoculated with TuMV::6K2-GFP. *Syp71* or *Vap27-1* expression was reduced in the VIGS vector treated plants to the level similar to the treated *Arabidopsis*, and the *Syp71*-downregulated plants showed resistance to TuMV (data not shown). By confocal laser microscopy, strong GFP signals were evident in the newly developed systemic leaves of the control plants (mock treated or infiltrated with the TRV empty vector) and of *Vap27-1*-downregulated plants but not detectable in those of *Syp71*-downregulated plants. In the inoculated leaves of the TRV empty vector-infected or *Vap27-1*-downregulated plants, 6K2-induced tubular structures and chloroplast aggregates occurred in a manner similar to those found in the wild type plants infected by TuMV::6K2-GFP (Fig. 6A). However, in the *Syp71*-downregulated plants, the formation of 6K2-induced plates and chloroplast aggregates was blocked, though the trafficking of 6K2-GFP to the periphery of chloroplasts was apparently not affected (Fig. 6A). These results suggest that Syp71 but not Vap27-1 is required for the formation of the elongated tubular structures and chloroplast clumps.

To further determine if inhibition of TuMV infection in *Syp71*-donwregulated *Arabidopsis* plants is due to reduced TuMV replication, we transformed the TuMV::6K2:GFP infectious clone into protoplasts isolated from WT or *Syp71*-downregulated *Arabidopsis* leaves and then assayed TuMV accumulation. Virus accumulation was significantly reduced in the *Syp71*-downregulated protoplasts at three different time points (Fig. 6B), suggesting Syp71 is essential for TuMV accumulation.

## Discussion

In a recent report, we established that plant potyviruses initiate viral genome translation on the ER and induces the formation of the 6K2 vesicles at the ER subdomain that subsequently traffic to chloroplasts to generate the chloroplast-6K2 vesicle complex for potyvirus replication [12]. More recently, Grangeon *et al.* localized the virus-induced complex containing the 6K2, chloroplasts as well as ER and Golgi markers into the perinuclear region [26]. In the present study, we observed that TuMV infection caused the formation of chloroplast clumps and elongated tubular structures at the junctions between neighbouring chloroplasts in more than 85% of infected *N. benthamiana* cells at 96 hpi (Fig. 1A, frames III–VI). This is in agreement with an earlier observation that chloroplast aggregates and elongated tubular structures between adjacent chloroplasts of the clump were present in TuMV-infected *Chenopodium quinoa* leaves [21]. Intriguingly, we also observed that about 30% of the infected cells showed the virus-induced perinuclear structure at 96 hpi (data not shown) as described [26], all containing the chloroplast aggregates. Since the chloroplast and 6K2 interaction was observed first, followed by chloroplast aggregation and then the perinuclear structure, it seems that the chloroplast-6K2 complex leads to chloroplast aggregation and further to the formation of the perinuclear structure.

In this study, we demonstrated that expression of the 6K2 protein alone is sufficient to induce the formation of chloroplast aggregates as well as elongated plates at the junctions between adjacent chloroplasts (Fig. 1B, frames II–VIII). As this phenomenon was not observed when other viral proteins including P1, HC-Pro, P3, P3N-PIPO, 6K1, CI, NIa-VPg, NIa-Pro, NIb and CP were expressed alone, it is reasonable to propose that it is the 6K2 protein that accounts for the generation of chloroplast aggregation and elongated tubular structures during potyviral infection. Infections by other plant positive single-stranded viruses can also induce similar cytopathological effects. For instance, the clumping of the chloroplasts is one of the typical cytological effects of *Turnip yellow mosaic virus* (TYMV) infection [27]. TYMV is a rod-shaped small RNA virus in the genius *Tymovirus* of the family *Tymoviridae*. TYMV replication complexes colocalize with virus-induced membrane vesicles that are thought to result from the invagination of the chloroplast envelope [28]. The TYMV 140K protein was shown to target the chloroplast envelope, develop vesicles along the chloroplast peripheries and finally induces the clumping of the chloroplasts [28]. As these vesicles contain TYMV viral replication complexes and viral particles, they have been suggested to be the TYMV replication site [27]. In contrast, TuMV sequentially recruits the ER and chloroplasts for its genome replication [12]. In TuMV:6K2-GFP infected *N. benthamiana* cells, double strand RNAs, a hallmark of viral genome replication, and the viral replicase components such as viral RNA-dependent RNA polymerase (also NIb) colocalize with chloroplast-bound 6K2 vesicles [12]. But no TYMV-type invaginations were found in chloroplasts [12], suggesting a different role of chloroplasts in TuMV infection. In TuMV-infected *Chenopodium quinoa* leaves, viral particles were found in the elongated tubular structure between clumped chloroplasts [21]. In the case of other potyviruses, chloroplast-bound elongated tubular structures and vesicles containing viral particles were also evident in *Wheat streak mosaic virus*-infected wheat leaf tissues [29]. In comparison with the highly dynamic ER, relatively stationary chloroplasts and chloroplast clumps may provide replicating potyviruses with an energy- and resource-rich environment effectively protecting them against host defense responses such as virus induced RNA silencing. Taken together, these data support the notion that the 6K2-induced chloroplast-bound tubular structures are the site for TuMV replication and viral particle assembly.

We identified a SNARE protein Syp71 and a SNARE-like protein Vap27-1 that were recruited to the 6K2-induced chloroplast-bound elongated tubular structure (Fig. 3 C–H). As TuMV infection was inhibited only in S*yp71*-downregulated plants (Fig. 5A) but not in *Vap27-1*-downregulated plants (Fig. 5B), Syp71 rather than Vap27-1 plays an essential role in the infection process. We further determined that inhibition of virus infection was due to reduced virus replication (Fig. 6B), highlighting the essential role of Syp71 in TuMV accumulation. Knockdown of *Syp1* expression blocked the formation of elongated tubular structures and chloroplast aggregates as well as the virus-induced perinuclear structure, but did not impair the trafficking of 6K2 vesicles to the outer chloroplast envelope (Fig. 6A), suggesting that Syp71 is involved in mediating the association of 6K2 vesicles with the outer chloroplast envelope and further homotypic fusion of 6K2 vesicles to assemble the elongated tubular structures and to join 6K2-bound chloroplasts. Indeed, SNARE family proteins are known for their essential role in specific membrane fusions between the transport vesicles and target membranes [14], [15]. Syp71, together with Syp72 and Syp73, forms a plant-specific SNARE subfamily named Syp7 [30]. Syp71 localizes to the plasma membrane and the ER in *Arabidopsis* [30]. As no homozygous T-DNA insertion *Arabidopsis Syp71* mutant could be isolated from the *Syp71*/*syp71* heterozygote, Syp71 seems essential for the development of *Arabidopsis*. To date no specific functions have been identified for the Syp7 subfamily except for a recent report showing that Syp71 is essential for symbiotic nitrogen fixation in *Lotus japonicas* nodules in a yet-unknown manner [31]. To our best knowledge, the current study represents the first to functionally characterize a SNARE family protein essential for virus infection in plants.

To explore how Syp71 is recruited to the 6K2 elongated tubular structure, we carried out a yeast two-hybrid assay followed by coimmunoprecipitation experiments to confirm protein-protein interactions *in planta*. Unexpectedly, Syp71 did not interact with the 6K2 protein (Fig. 4A), suggesting that the recruitment of Syp71 to the 6K2 elongated tubular structure is via an indirect approach. Since Vap27-1 interacted with both 6K2 and Syp71 (Fig. 4A), it is possible that Vap27-1 may function as a linker between the 6K2 vesicle and Syp71. This assumption was supported by our coimmunoprecipitation assay showing that 6K2, Vap27-1 and Syp71 indeed can be coimmunoprecipitated (Fig. 4D). Consistently, it is well known that the SNARE-like VAP protein such as Vap27-1 sharing high homology with the mammalian VAP33 can regulate the trafficking, docking and fusion of vesicles through their interactions with SNARE proteins [18], [32], [33]. Previously Vap27-1 was shown to colocalize with membranous vesicles induced by the 60K membrane protein of CPMV and to interact with the CPMV 60K protein, and was proposed to play a role in the induction of membrane vesicles and/or the assembly of the replication complex [9]. In this study, knockdown of *Vap27-1* expression did not affect the development of elongated tubular structures and chloroplast aggregates in TuMV-infected cells and did not significantly affect virus accumulation (Fig. 5B). These results suggest an alternative recruitment approach for Syp71 to the TuMV chloroplast-associated vesicles. It is possible that other VAPs or proteins that have overlapping functions with Vap27-1 interact with 6K2 and Syp71 and mediate the association of Syp71 with the 6K2 vesicles. Search for the other components of the Syp71 or 6K2 interactome via diverse proteomic tools including yeast two-hybrid screens with Syp71 or 6K2 as bait will certainly help to elucidate the mechanism underlying the formation of chloroplast-bound 6K2 elongated tubular structures and shed new insights into specific host-virus interactions in the infection process.

## Materials and Methods

### Gene cloning, plasmid construction and transient expression in *N. benthamiana*

Gateway technology (Invitrogen) except otherwise stated was used to generate clones reported in this study. RNA extraction from *Arabidopsis thaliana* or *Nicotiana benthamiana* leaves was performed using an RNeasy Plant Mini Kit (Qiagen). Reverse transcription was catalyzed by Superscript III reverse transcriptase (Invitrogen) with oligonucleotide dT20. Gene sequences were amplified by PCR using Phusion DNA polymerase (NEB). The resulting DNA fragments were purified and transferred by recombination into the entry vector pDONR201 (Invitrogen) using BP clonase II (Invitrogen) following the manufacturer's protocol. The insert of the resulting pDONR clone was verified by sequencing. The insert was then transferred by recombination to the indicated binary destination vector using LR clonase II (Invitrogen) following the standard conditions and procedure recommended by the supplier. SNAREs proteins Syp71 (At3g09740), Syp72 (At3g45280), Syp73 (At3g61450), Syp81 (At1g51740), VAM723 (At2g33110), Vap27-1(AY364005) and Vap27-2 (AY364004.1) coding sequences were amplified from cDNA derived from *Arabidopsis* RNA and recombined into pDONR201. The inserts of these resulting intermediate clones were further transferred by recombination into the binary destination vectors pEarleygate101 [34] to generate plasmids Syp71-YFP, Syp72-YFP, Syp73-YFP, Syp81-YFP, VAMP723-YFP, Vap27-1-YFP and Vap27-2-YFP, as well as into the binary destination vectors pEarleygate101 and 103 to generate Syp71-HA and Vap27-1-His, respectively. The recombinant TuMV infectious clones TuMV-GFP (EF028235.1) consisting of a full-length TuMV cDNA tagged by GFP and TuMV::6K2-GFP containing an additional copy of 6K2 fused to GFP were described previously [35]. The 6K2 cistron of TuMV was amplified by PCR. Gateway technology with the entry vector pDONR201 and destination vectors pEarleygate101 and pEarleygate102 was again used to produce plasmids 6K2-HA-YFP and 6K2-CFP, respectively. The plasmids including mTalin-CFP containing the mouse talin (mTalin) sequence [36], [37] was described previously. The plasmids containing the tails of myosin XI-K and XI-2 from *N. benthamiana* were described previously [24]. The full-length of TuMV 6K2 sequence was cloned from plasmid TuMV-GFP and engineered into the bait vector pBT3-STE (Dualsystems Biotech, Schlieren, Switzerland) using the *Sfi*I restriction site, designated as pBT3-STE-6K2. The complete open reading frame coding for the Syp71 (conserved Syp71 domain in sequence accession number AT3G09740) and Vap27-1 (accession number AY364005) proteins were amplified by PCR from wild type *Arabidopsis* cDNA, inserted into pBT3-STE and pPR3-N vectors (Dualsystems Biotech ) using the *Sfi*I restriction site and designated as pPR3-N-Syp71, pPR3-N-Vap27-1, and pBT3-STE-Vap27-1.

Four-week-old *N. benthamiana* plants grown in a greenhouse at 22°C to 24°C were used for *Agrobacterium tumefaciens* (strain GV3101)-mediated transient expression as described previously [12], [38], [39].

### Chloroplast preparation and western blots

Chloroplasts were isolated from infected *N. benthamiana* leaves essentially as described [12]. Immunoblotting was carried out with rabbit sera raised against *Arabidopsis* Syp71 or against *Arabidopsis* Vap27-1 as described [40], [41]. Chloroplast purity was monitored by immunoblotting with antisera against the chloroplast marker PsbA and the ER marker Bip2.

### Confocal microscopy

Confocal microscopy and subsequent image processing were conducted essentially as described [42]. Plant tissues were imaged at room temperature using a Leica TCS SP2 inverted confocal microscope with a 63 oil immersion objective. For confocal microscopy, a UV laser and a krypton/argon laser were used to examine fluorescence. GFP was excited at 488 nm, and the emitted light was captured at 497 to 510 nm; CFP was excited at 405 nm, and the emitted light was captured at 440 to 470 nm; YFP was excited at 514 nm, and the emitted light was captured at 525 to 650 nm. Images were captured digitally and handled using the Leica LCS software. Post-acquisition image processing was done with Adobe Photoshop 5.0 software.

### Yeast two-hybrid assay

Yeast transformation were performed using the lithium acetate-based protocol for preparing and transforming yeast competent cells following the instructions of the DUAL membrane pairwise interaction kit user manual (Dualsystems Biotech). This system provides bait vectors, prey vectors and controls to perform pairwise interaction assays between membrane proteins. In brief, bait and prey vectors were described above. Different pairs of resulting plasmids were cotransformed into yeast strain NMY51 (*MATa his3 trp1 leu2 ade2 LYS2::HIS3 ura3::lacZ ade2::ADE2 GAL4*) using the DS Yeast transformation kit (Dualsystems Biotech). Transformed colonies were selected in SD-LW medium and incubated for growth of positive transformants. For growth assays, independent positive transformants were selected and grown in SD-LW liquid medium at 30°C overnight. Culture concentrations were adjusted at OD546 = 1 and diluted 10, 100 and 1000 times. Three microlitres of each dilution was spotted on to SD-LW and SD-AHLW solid media, respectively, and incubated at 30°C for 2 days.

### Immunoprecipitation

After *Agrobacterium*-mediated transient expression for 48 h, *N. benthamiana* leaves (approximately 0.3 g) were harvested, and ground to powder in liquid nitrogen. Ground tissues were resuspended in 3.0 mL of IP buffer containing 50 mM Tris, pH 7.5, 150 mM NaCl, 10% glycerol, 0.1% Nonidet P-40, 5 mM dithiothreitol, and 1.5× Complete Protease Inhibitor (Roche). The crude lysates were then spun at 20,000 g for 15 min at 4°C to precipitate tissues, cells and cell debris. After centrifugation, 1 ml of supernatant was incubated with 0.5 mg of the indicated monoclonal antibody for each immunoprecipitation. After incubation for one hr at 4°C, immunocomplexes were collected by the addition of 50 mL of protein G Sepharose-4 fast flow beads (Amersham) and incubated for 4 h at 4°C. The immunocomplexes were washed four times with 1 ml of IP buffer and the pellet was resuspended in 3×SDS-PAGE loading buffer (12). The obtained protein samples were separated by SDS-PAGE on 12% polyacrylamide gels and transferred by electroblotting to nitrocellulose membranes. Membranes were probed with anti-HA horseradish peroxidase (Sigma) or anti-HIS peroxidase (Abcam) to detect HA- and HIS-epitope-tagged proteins, respectively. All immunoprecipitation experiments were repeated three times.

### TRV-based gene silencing

For TRV-based gene silencing, four pairs of primers AtSyp71F/AtSyp71R, AtVap271F/AtVap271R, NbSyp71F/NbSyp71R, and NbVap27F/NbVap27R were used to amplify *Arabidopsis* and *N. benthamiana* leaf cDNAs to generate partial cDNA sequences of *Syp71* and *Vap27-1* from *Arabidopsis* and *N. benthamiana*. The resulting cDNA fragments were digested with *EcoR*I and *BamH*I and then ligated into the corresponding sites of TRV2 [43]. To silence *Syp71*, TRV1 and TRV2-AtSyp71 (or TRV2-NbSyp71) were coagroinfiltrated into *Arabidopsis* (or *N. benthamiana*) essentially as described [43]. Similarly TRV1 and TRV2-Vap27-1 (or TRV2-Nb271) were coagroinfiltrated to silence *Vap27-1* in *Arabidopsis* (or *N. benthamiana*). Ten days post-infiltration, treated seedlings were inoculated with TuMV::6K2-GFP.

### Real-time quantitative RT-PCR

Total RNA isolation and DNAse I treatment were as described above. RT reactions were performed with SuperScript III First-Strand Synthesis System for RT-PCR kit (Invitrogen) according to the manufacturer's instructions. qPCR was performed using the CFX96 real-time PCR system (BioRad) following the manufacturer's instructions. Relative amounts of all mRNAs were calculated from threshold cycle values. The *ActinII* reference gene (ACT2F: 5′- GCCATCCAAGC TGTTCTCTC- 3′ and ACT2R: 5′- GAACCACCGATCCAGACACT-3′) was used for normalization. Primers CP-F (5′-TGGCTGATTACGAACTGACG-3′) and CP-R (5′-CTGCCTAAATGTGGGTTTGG-3′) were used for TuMV detection. All results were shown as means of at least three biological replicates with corresponding standard errors.

### Protoplast isolation and TuMV replication assay

Wild type and *Syp71*-downregulated *Arabidopsis* leaves treated with TRV-based gene silencing (10 dpi) were used for isolation of mesophyll protoplasts following an established protocol [44]. TuMV replication assay was carried out essentially as described [45]. Briefly, protoplasts (about 1 million) were transfected with 0.5 µg of TuMV::6K2-GFP plasmid in the presence of 18% PEG 400 for 10 min. Transfected protoplasts were washed and resuspended in W5 buffer. The transformed protoplasts were then incubated at RT and harvested at planned time points. Viral RNA accumulation was determined by qRT-PCR as described above.
